# Establishment of Insulin-Producing Cells From Human Embryonic Stem Cells Underhypoxic Condition for Cell Based Therapy

**DOI:** 10.3389/fcell.2018.00049

**Published:** 2018-05-15

**Authors:** Piyaporn Rattananinsruang, Chavaboon Dechsukhum, Wilairat Leeanansaksiri

**Affiliations:** ^1^School of Preclinic, Institute of Science, Suranaree University of Technology, Nakhon Ratchasima, Thailand; ^2^School of Pathology, Institute of Medicine, Suranaree University of Technology, Nakhon Ratchasima, Thailand

**Keywords:** human embryonic stem cells, differentiation, insulin-producing cells, hypoxic condition, diabetes

## Abstract

Diabetes mellitus (DM) is a group of diseases characterized by abnormally high levels of glucose in the blood stream. In developing a potential therapy for diabetic patients, pancreatic cells transplantation has drawn great attention. However, the hinder of cell transplantation for diabetes treatment is insufficient sources of insulin-producing cells. Therefore, new cell based therapy need to be developed. In this regard, human embryonic stem cells (hESCs) may serve as good candidates for this based on their capability of differentiation into various cell types. In this study, we designed a new differentiation protocol that can generate hESC-derived insulin-producing cells (hES-DIPCs) in a hypoxic condition. We also emphasized on the induction of definitive endoderm during embryoid bodies (EBs) formation. After induction of hESCs differentiation into insulin-producing cells (IPCs), the cells obtained from the cultures exhibited pancreas-related genes such as *Pdx1, Ngn3, Nkx6.1, GLUT2*, and *insulin*. These cells also showed positive for DTZ-stained cellular clusters and contained ability of insulin secretion in a glucose-dependent manner. After achievement to generated functional hES-DIPCs *in vitro*, some of the hES-DIPCs were then encapsulated named encapsulated hES-DIPCs. The data showed that the encapsulated cells could possess the function of insulin secretion in a time-dependent manner. The hES-DIPCs and encapsulated hES-DIPCs were then separately transplanted into STZ-induced diabetic mice. The findings showed the significant blood glucose levels regulation capacity and declination of IL-1β concentration in all transplanted mice. These results indicated that both hES-DIPCs and encapsulated hES-DIPCs contained the ability to sustain hyperglycemia condition as well as decrease inflammatory cytokine level *in vivo*. The findings of this study may apply for generation of a large number of hES-DIPCs *in vitro*. In addition, the implication of this work is therapeutic value in type I diabetes treatment in the future. The application for type II diabetes treatment remain to be investigated.

## Introduction

Diabetes mellitus (DM) is a chronic metabolic disease associated with the manifestation of high blood glucose levels. Type I diabetes (insulin-dependent diabetes) results from the cellular mediated autoimmune destruction of pancreatic islet cells leading to absence of insulin. Type II diabetes causes by abnormal functions of insulin hormone. The high blood glucose level could increase the risk of diabetes complications. The patients will have serious consequence problems in so many parts of the body such as neuropathy, nephropathy, retinopathy and heart disease (Cheung and Wong, 2008). At present, islet cell transplantation is a current successful treatment in a highly selected population of type I diabetic patients with the Edmonton regimen (Vantyghem et al., 2009; Matsumoto, 2016; Jin and Kim, 2017). Subsequent treatment showed that transplanted cells could potentially reversal of diabetes. Although the feasibility of this curative therapy is the prevention of the diabetes complications (Brennan et al., 2016), the insufficient islets supply remains a critical issue for the success of islet transplantation for diabetes treatment (Noguchi, 2009; McCall and Shapiro, 2012). Therefore, the requirement for an effectively unlimited supply for pancreatic β-cell replacement has led to explore the way to generate insulin-producing cells (IPCs) from stem cells for therapeutic application of this disease treatment.

Human embryonic stem cells (hESCs) represent a promising tool for generating IPCs due to their differentiation capability into variety cell types. In addition, hESCs exhibit self-renewal capacity and can proliferate for a prolong period of time (Pera and Trounson, 2004; Teo and Vallier, 2010; Lilly et al., 2016). Therefore, the derivation of IPCs from hESCs may generate a large number of cells for therapeutic use (Van Hoof et al., 2009; Hebrok, 2012; Ahmed and Sayed, 2016). Regarding the controlled generation of functional IPCs from hESCs, there are still many challenges related to establish the efficient differentiation protocol. The generation of IPCs from hESCs is a complex process that involves the certain classes of growth factors, resulting in the pattern resembling pancreatic development *in vivo*. There is evidence that the IPCs develop in the cultures displays a heterogeneous mixture of undifferentiated hESCs, IPCs and differentiated cells from other lineages (Jiang et al., 2007; Raikwar and Zavazava, 2009; Wei et al., 2013). Moreover, oxygen tension is an important microenvironmental factor that may influence the differentiation state of hESCs. It has been documented that different oxygen conditions may affect many cellular responses. Culture condition for maintaining hESCs in an atmospheric (21% O_2_) or normoxic has been induced spontaneous cell differentiation. On the other hand, a physiologic (2–5% O_2_) or hypoxic condition has been shown to maintain hESC pluripotency and proliferation (Forristal et al., 2010; Närvä et al., 2013). It is noteworthy that hypoxic stress increases the differentiation of hESCs into desired cell types via embryoid bodies (EBs) intermediate stage (Niebruegge et al., 2009; Horton and Auguste, 2012). Moreover, hypoxic preconditioning represents a clinically applicable regime to improve graft survival and the therapeutic potential of hESC transplantation (Francis and Wei, 2010). These observations suggest that oxygen tensions are critical factor in successful hESC bioprocessing.

In this study, we generated a new protocol to establish IPCs from hESCs referred hES-DIPCs in hypoxic condition. The study also provides information regarding pancreas-related gene expression and insulin secretion in glucose dependent manner of hES-DIPCs. After achievement to generated functional hES-DIPCs *in vitro*, about half of the hES-DIPCs were then encapsulated to become encapsulated human embryonic derived insulin producing cells (encapsulated hES-DIPCs) and subjected to further functional investigations *in vivo* a long with none encapsulated cells (hES-DIPCs). The findings of this study may apply for generation of a large number of hES-DIPCs *in vitro*. In addition, the achievement of maintaining blood glucose level to prevent hyperglycemia condition as well as decrease inflammatory cytokine level *in vivo* of both hES-DIPCs and encapsulated hES-DIPCs exhibits therapeutic value in type I and type II diabetes treatment in the future.

## Materials and methods

### Culture of undifferentiated hESCs

The hESCs line H9 (Wicell Research Institute, Madison, USA) was maintained in the undifferentiated state by culture on the layer of mytomycin-C treated human forskin fibroblast (hFF) feeder. Undifferentiated hESCs were grown in hESC medium containing 79% knockout Dulbecco's modified Eagle's medium (KO-DMEM), 20% knockout serum replacement (KO-SR), 1% non-essential amino acid, 1 mM L-glutamine, 0.1 mM β-mercaptoethanol and 5 ng/ml basic fibroblast growth factor (bFGF) at 37°C, 5% O_2_, 4.5% CO_2_ and 95% humidity. The cells were passage every 5–7 days.

### Formation of EBs

Undifferentiated hESC colonies were mechanically dissecting into pieces less than 200 μm in size. The hESC pieces were cultured in the absence of feeder layers in “hanging drops” (one pieces/ 20 μl drop) to produce aggregates called “EBs” for 2 days in hESC culture medium without bFGF. At day 3, EBs were transferred into 6-well ultra-low attachment culture plate (Corning, Lowell, MA) and cultivated for 5 days in culture medium consisted of hESC medium (without bFGF) supplemented with 100 ng/ml activin A (Peprotech) to accelerate more endoderm layer formation of the EBs. Cells were grown in 37°C, 5% O_2_, 4.5% CO_2_ and 95% humidity.

### *In vitro* differentiation of IPCs

For IPCs differentiation, EBs were cultivated further in attachment culture condition (0.1% gelatin coated-35 mm tissue culture dish) and cultured for 14 days in the medium mainly composed of KO-DMEM containing 2% B27 (Invitrogen), 2 ng/ml bFGF, 20 ng/ml EGF (Peprotech), 100 ng/ml noggin (Peprotech) and 10 ng/ml betacellulin (Peprotech) **(**Stage 1**)**. Then, the differentiated cells were cultured in culturing medium as stage 1 but in the absence of bFGF for 7 days **(**Stage 2). At day 29, the cells were cultured in a maturation medium is defined as KO-DMEM plus 10 mM nicotinamide (Sigma-Aldrich), 50 ng/ml IGF II (Peprotech), 10 ng/ml betacellulin and 50 ng/ml HGF (Peprotech) to generate IPCs for 18 days **(**Stage 3). These differentiated cells were incubated at 37°C, 5% O_2_, 4.5% CO_2_ and 95% humidity. The differentiation media were changed every 3 days at all stages.

### Quantitative real-time polymerase chain reaction (PCR)

Undifferentiated hESCs, EBs and differentiated stage 1–3 cells were collected. RNA was extracted using RT100 Total RNA Mini kit (Geneaid). RNA concentrations were measured by using the NanoDrop ND-100 Spectrophotometer (NanoDrop Technologies Inc.) and 50-100 ng of this RNA was used in a reverse transcription (RT) reaction with a cDNA Synthesis kit (Fermentas). Real-time PCR was carried out with SYBR Green master mix (Applied Biosystems) using forward and reverse primers (listed in Table 1). The reaction was performed in an ABI 7900HT real time PCR system (Applied Biosytems). The relative expression values were normalized relative to the housekeeping gene GAPDH and the values from the differentiated cells samples were compared to those of the undifferentiated hESCs.

**Table 1:** Primer sequences and PCR conditions used in the real-time PCR.

**Gene**	**Primer Sequence (5'−3')**	**Annealing Temp. (°C)**	**Size (bp)**	**References**
*GAPDH*	F: AGCCACATCGCTCAGACACC R: GTACTCAGCGGCCAGCATCG	60	302	Yao et al., 2006
*Nestin*	F: CAGCTGGCGCACCTCAAGATG R: AGGGAAGTTGGGCTCAGGACTGG	55	208	Shim et al., 2007
*Pdx1*	F: CCCATGGATGAAGTCTACC R: GTCCTCCTCCTTTTTCCAC	58	206	Seeberger et al., 2006
*Hnf3β*	F: CCACCACCAACCCCACAAAATG R: TGCAACACCGTCTCCCCAAAGT	60	294	Baharvand et al., 2006
*Ngn3*	F: GGTAGAAAGGATGACGCCTC R: CCGAGTTGAGGTCGTGCAT	58	313	Seeberger et al., 2006
*NeuroD1*	F: GCCCCAGGGTTATGAGACTATCACT R: CCGACAGAGCCCAGATGTAGTTCTT	61	523	Khoo et al., 2005
*Nkx6.1*	F: GTTCCTCCTCCTCCTCTTCCTC R: AAGATCTGCTGTCCGGAAAAAG	53	381	Segev et al., 2004
*GLUT2*	F: AGGACTTCTGTGGACCTTATGTG R: GTTCATGTCAAAAAGCAGGG	55	231	Segev et al., 2004
*Insulin*	F: CTACCTAGTGTGCGGGGAAC R: CACAATGCCACGCTTCTG	60	~150	Yu et al., 2007

### Immunofluorescence assay

At the end of differentiation, cells were fixed in 4% paraformaldehyde in phosphate buffer saline (PBS) and blocked with 3% serum and 0.2% triton X-100 in PBS. The cells were then incubated overnight at 4°C with primary antibody. After washed with PBS, cells were incubated with secondary antibody for 1 h at room temperature. The cells were counterstained nuclei with 4,6-diamidino-2-phenylindole (DAPI) (Invitrogen) for 3 min at room temperature. Finally, the cells were mounted with mounting media (VECTASHIELD, Vector Labs, Burlingame, CA). The immunofluorescence images were analyzed using a confocal microscope (Olympus American Inc. Melville, NY).

The following primary antibodies and dilutions were used; mouse anti-hNestin 1:100 (R&D Systems, MAB1259); guinea pig anti-insulin, 1:100 (Dako, A0564); rabbit anti-glucagon, 1:100 (Dako, A0565); mouse anti-human pro-insulin c-peptide 1:100 (Chemicon, CBL94); mouse anti-somatostatin (Santa Cruz Biotechnology, SC-25262). Secondary antibodies were FITC-conjugated goat anti-mouse 1:100 (BD Pharmingen), FITC-conjugated swine anti-rabbit (Dako) and Cy3-conjugated goat anti-mouse 1:100 (Jackson Immuno Research Labs).

### Dithizone (DTZ) staining

A DTZ (Sigma) stock solution was prepared with 50 mg of DTZ in 5 ml of dimethylsulfoxide (DMSO) and store briefly at −15°C. *In vitro* DTZ staining was performed by adding 20 μl of the stock solution to 1 ml of culture medium. Then, the cells were incubated at 37°C for 15 min. After rinsed with Hank's balanced salt solution (HBSS), the stained cells were analyzed by a phase contrast microscope. The Dithizone (DTZ) is a zinc-binding substance which can mark the beta cells containing Zinc within the cells. The pancreatic islets which are positive with this staining (red color by stained with crimson red in the solution) account for the achievement of hESCs differentiation into beta cells or insulin producing cells.

### Measurement of insulin secretion of differentiated cells

The differentiated cells at stage 3 were rinsed twice in Krebs-Ringer bicarbonate HEPES (KRBH) buffer. The cells were then incubated in KRBH buffer containing 5, 20, and 50 mM glucose at 37°C for 1 h, respectively. Supernatant were collected for insulin secretion measurement. Insulin levels were determined by insulin enzyme-linked immunosorbent assay (ELISA) kit (Dako). The hES-IPCs population that can secrete insulin in a glucose dependent manner were separate into 2 parts. One part subjected for alginate encapsulation and another part remain non-encapsulation.

### Alginate encapsulation of hES-DIPCs

Part of the hES-DIPCs population that can secrete insulin in a glucose dependent manner were suspended in a 1.5% alginate solution at a concentration of 40,000 cells/ml. Alginate beads were generated by extruding the cell-alginate mixture from plastic syringe via 27G needle into a 200 ml bath of CaCl_2_ (100 mM), containing 145 mM NaCl, and 10 mM MOPS. Formed beads were left in CaCl_2_ bath for 10 min at room temperature for polymerization to occur. The CaCl_2_ bath was continuously agitated by a magnetic stirrer to avoid coalescence of bead during polymerization. Following the encapsulation step, the beads were transferred to a tissue culture 6-well plate. The CaCl_2_ solution was removed and beads were washed in washing buffer solution. Finally, washing buffer solution was removed and then cultured overnight in complete medium at 37°C.

### *In vitro* assessment of insulin secretion of encapsulated hES-DIPCs

The encapsulated hES-DIPCs were cultured at 37°C, 5% O_2_, 4.5% CO_2_ and 95% humidity. Media samples were collected at day 0, 2, 4, 6, 8, 10, 12, and 14 to assess the rate of insulin secreted by the encapsulated cultures. Insulin levels were determined by insulin enzyme-linked immunosorbent assay (ELISA) kit (Dako).

### Transplantations of hES-DIPCs and encapsulated hES-DIPCs into diabetic mice

Mice and the animal works in this study were conducted in accordance with the Ethical Principles and Guidelines for the Use of Animals (National Research Council of Thailand) and the SUT laboratory animal use monitoring committee (Suranaree University of Technology, SUT). Adult male mice weighing approximately 20–30 g bred were used. The animals were maintained under controlled temperature (23–25°C) with a 12 h light and 12 h dark lighting. They were fed with a water and standard laboratory diet *ad libitum* throughout the experiment.

To generate diabetic mice, Streptozotocin (STZ) was freshly dissolved in cold 0.1 M citrate buffer (pH 4.5) before intraperitoneal injection into prepared mice which fasted for overnight (12 h). All mice received STZ in the dose of 40 mg/kg body weight for 5 consecutive days. To overcome the drug-induced hypoglycemia, the mice were allowed to drink 2% glucose solution for 2 days. Control mice were injected with 0.1 M citrate buffer (pH 4.5) alone. Diabetes was confirmed by the determination of fasting glucose concentration on the seventh day post-administration of STZ. STZ-treated animals were considered as diabetic when the blood glucose ranges above 250 mg/dl.

The diabetic mice were then divided into three groups (1) diabetic control mice (2) diabetic mice subcutaneously (SC) injected with hES-DIPCs (100,000 cells per one animal), and (3) diabetic mice SC injected with encapsulated hES-DIPCs. (100,000 cells corresponding to a capsule volume of 500 μl in a 1 ml syringe per animal). All treated mice did not receive any immunosuppression and the cells were injected at D0, D28, D42 and D56. The body weight was measured weekly during the experiment period.

### Assessment of *in vivo* transplantation

To determine the ability of hES-DIPCs and encapsulated hES-DIPCs functions in blood glucose level regulation, the transplanted and control mice were fasted overnight to determine the fasting blood sugar (FBS). Blood samples were then obtained from the tail bleeding every 14 days until 70 days post-transplantation. The FBS baseline of each mouse was determined prior to transplantation. The blood sugar levels were measured by Accu-Chek Advantage II (Roche Diagnostic, Mannheim, Germany). The body weight of experimental animals was also measured before and after transplantation.

### Detection of inflammatory mediators

To examine whether hES-DIPCs and encapsulated hES-DIPCs induce *in vivo* inflammation or not, blood of each transplanted animal was obtained. The measurement of Interleukin 1 beta (IL-1β) cytokine level in mouse serum was performed by using an enzyme immunoassay technique (Mouse IL-1β/IL-1F2: Quantikine, R&D systems). The cytokine contents were expressed as pg/ml. The mean minimum detectable dose (MDD) of mouse IL-1β was 2.31 pg/ml. The test was performed following the instruction of the manufacturer.

### Statistical analysis

The results expressed as mean ± SEM. Statistical analysis was performed using SPSS software package. All data were analyzed by using the one-way ANOVA followed by Duncan's test. The *P* < 0.05 was considered as significant.

## Results

### Generation of hESC-derived IPCs (hES-DIPCs) under hypoxic condition

**Figure 1:**
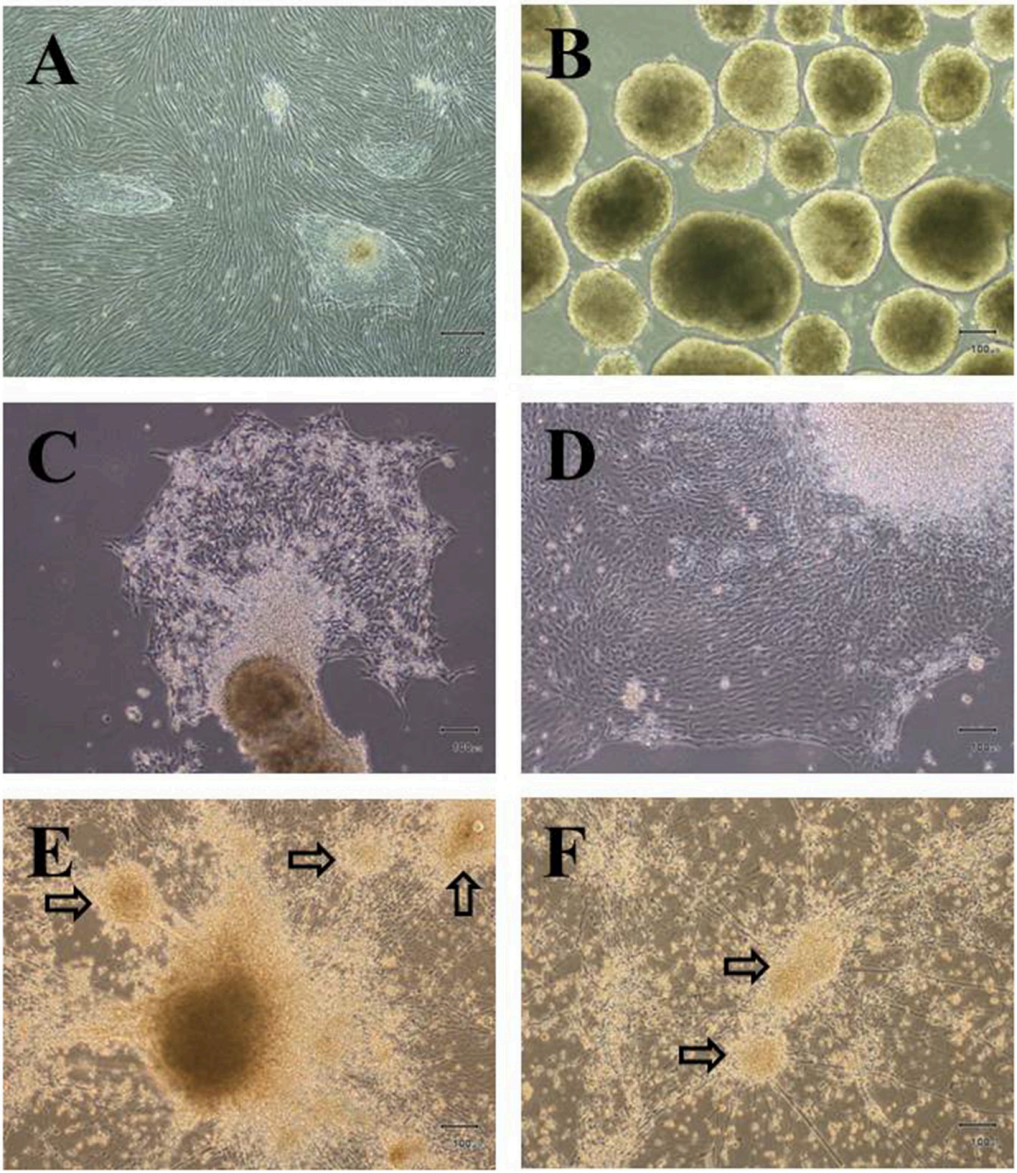
Differentiation of hESCs into insulin-producing cells. Phase contrast microscopy of undifferentiated hESC colonies **(A)**, Embryoid bodies; EBs **(B)** and differentiated cells at D21 **(C)**, D28 **(E)** and D46 **(E,F**), arrows indicate small islet-like cell clusters. Scale bars, 300 μm **(A**); 100 μm **(B–F**).

We have developed *in vitro* culture condition to differentiate hESCs into IPCs (hES-DIPCs) using several growth factors by modification of previously published protocols. We also decided to investigate whether the differentiation process in a hypoxic environment (5% O_2_) can further generate the hES-DIPCs. In order to promote the hES-DIPCs, we differentiated H9 hESCs (Figure 1A) into definitive endoderm (DE) committed-cells beginning with the formation of EBs in the presence of activin A. After an induction period of 7 days, the EBs were found as three dimensional ball structure (Figure 1B). After healthy EBs were obtained, the EBs were subsequently subjected to differentiation process, stage 1–3, as described in Materials and Methods section. In the stage 1 of differentiation, the EBs were cultured at a concentration of about 30 EBs per 0.1% gelatin-coated culture dish, in an induction medium containing KO-DMEM with bFGF, EGF, noggin and betacellulin. The EBs were attached and grown into a confluent monolayer by 8 days post-differentiation. The cells were allowed to further mature in this cocktail for 6 days for pancreatic endoderm induction (Figure 1C). At the end of 21 days period, the bFGF-supplemented was removed and the cells were allowed to differentiate further to day 28 (stage 2). In this stage, the cells underwent differentiation into pancreatic endocrine cells. The cell morphology was illustrated as Figure 1D. Cells from stage 2 were then cultured in stage 3 differentiation medium containing KO-DMEM with nicotinamide, IGFII, betacellulin and HGF for 18 days. In the last stage, these cells were indicated possible differentiation of cells toward IPCs. The formations of small islet cell clusters were obtained as demonstrated in Figures 1E,F, respectively.

### Characterization of hES-DIPCs

**Figure 2:**
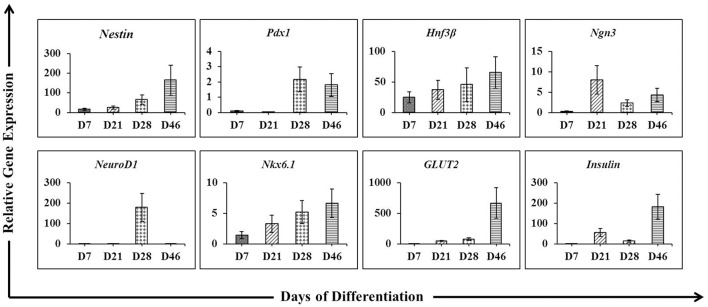
The expression pattern of pancreatic lineage genes under a protocol to differentiate hESCs into insulin-producing cells. Quantitative reverse transcription-polymerasse chain reaction (RT-PCR) was performed on days 7, 21, 28, and 46 of differentiation. The levels of *Nestin, Pdx1, Hnf3*β*, Ngn3, NeuroD1, Nkx6.1, GLUT2*, and *Insulin* gene expression were determined. For each sample, the relative gene expression levels were normalized to corresponding levels in undifferentiated hESCs.

To assess whether the differentiation protocol used in this experiment is an optimized protocol for differentiation of hESCs into IPCs. The differentiation of the cells was examined throughout the processes, including cells at day 7, 21, 28, and 46. The pancreatic genes including *Nestin, Pdx1, Hnf3*β, *Ngn3, NeuroD1, Nkx6.1, GLUT2*, and *insulin* were analyzed by real-time PCR analysis as demonstrated in Figure 2. The results showed that among the pancreatic genes, *Hnf3*β and *Nkx6.1* were expressed in the cells at D7 (EBs formation stage) and steadily increased until the final stage of differentiation. In these EBs, the other genes were absent or slightly expressed. *Nestin* was weakly expressed in the EBs, but progressively upregulated from D21 until D46 of differentiation process. The cells at D21 were highly expressed the endocrine progenitor marker *Ngn3*; however, the expression reduced along the progression of differentiation stage. The pancreatic transcription factor *Pdx1* was detected mainly in induced cells (D28) and minimal decreased in 46 days post-differentiation (D46). On the other hand, the expression of *neurogenic differentiation 1* (*NeuroD1*) showed maximal expression only at D28. Our analysis further demonstrates that the expression of *insulin* was highly induced at later stage of differentiation (D46). In addition, the glucose transporter *GLUT2* was also induced between D21 and D46 with a higher level at the end of the differentiation process (D46).

**Figure 3:**
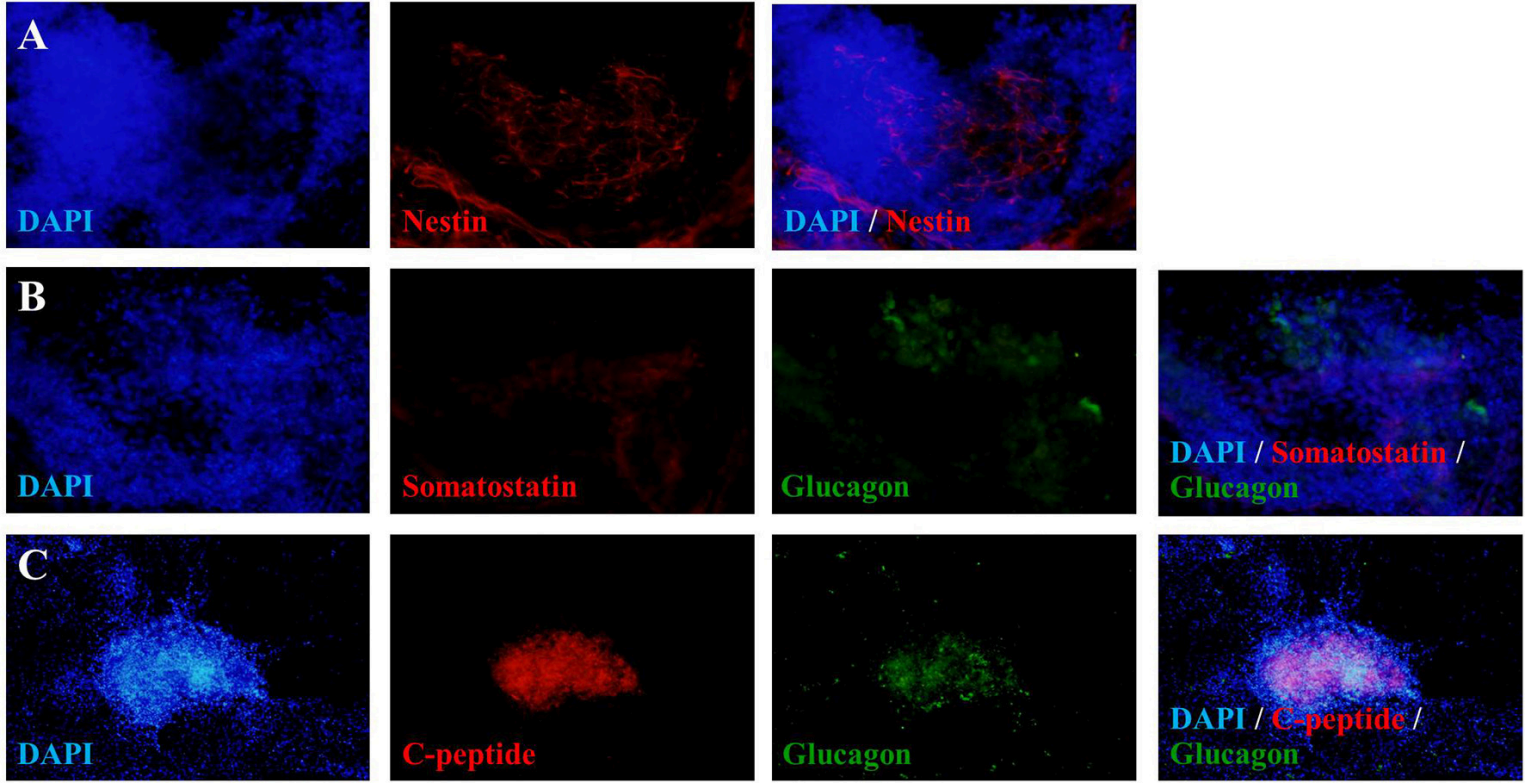
Fluorescence microscopic imaging of immunostaining for nestin, somatostatin, glucagon and C-peptide. The cells after 46 days of differentiation were stained with antibodies against nestin (red), somatostatin (red), glucagon (green) and C-peptide (red), followed by appropriate secondary antibodies conjugated to fluorochromes (blue, DAPI for nuclear staining; red, Cy-3; green, FITC). A merge of both images shows localization of nestin **(A)**, somatostatin **(B)**, glucagon **(B,C)**, and C-peptide **(C)**. The images were taken at magnifications of ×20 **(A,B)** and ×10 **(C)**.

To verify the achievement of hES-DIPCs induction, the protein expressions of nestin, glucagon, somatostatin and c-peptide (a byproduct of insulin production) were analyzed by immunofluorescence of D46 cells (Figures 3A–C). The data indicated that expression of C-peptide was highly expressed in these cells (Figure 3C). In this stage, the other pancreatic endocrine hormones including glucagon (Figures 3B,C), and somatostatin (Figure 3B) were also observed. However, slightly population of nestin-expressing cells was detected in day 46 of differentiation which represented the immature stage of the insulin producing cells (Figure 3A).

**Figure 4:**
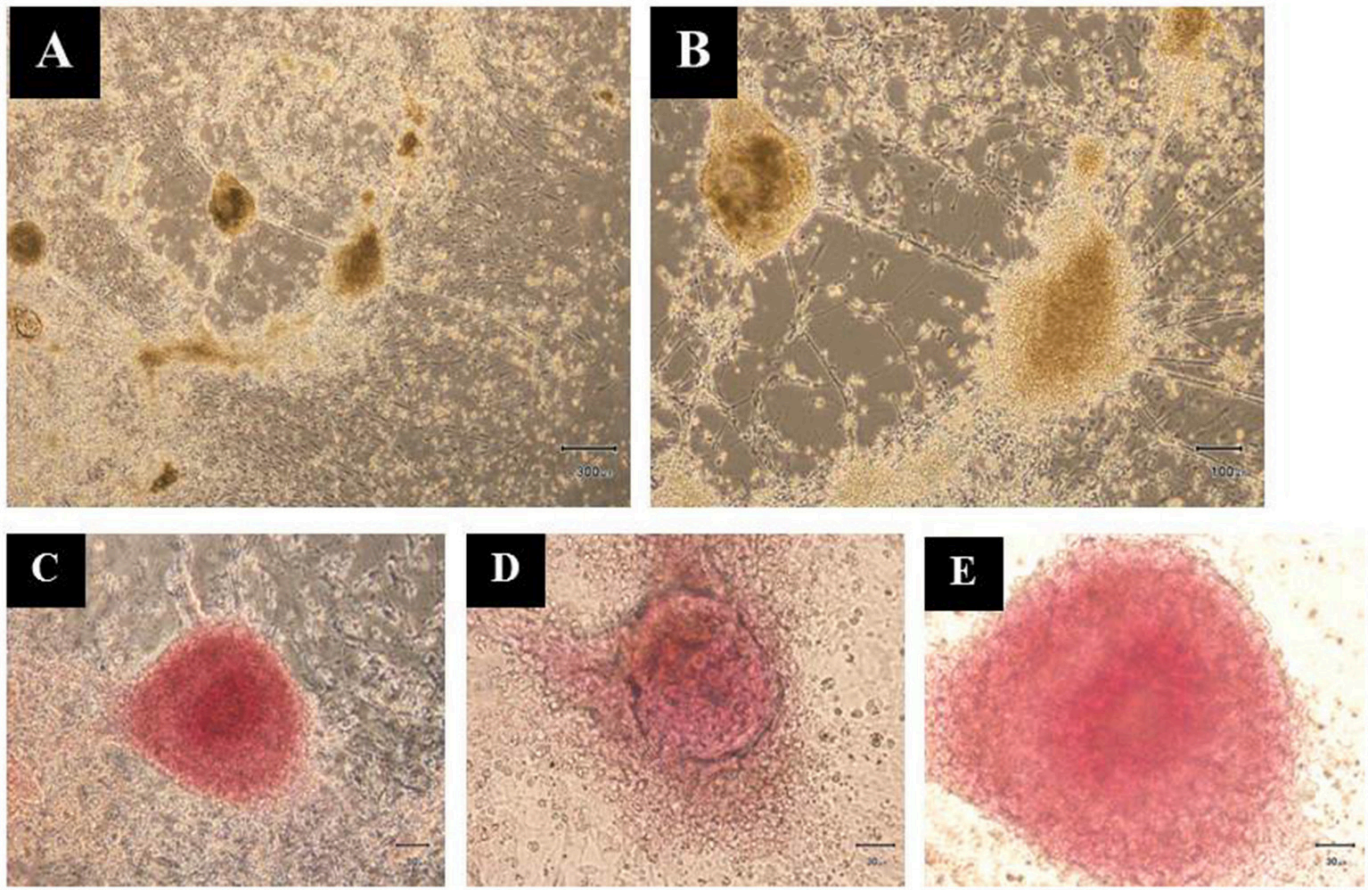
Dithizone (DTZ) staining. The cells after 46 days of differentiation were identified for insulin-producing cells within the differentiating hESCs **(A,B)**. These clusters were DTZ positive **(C–E)**. Scale bars, 300 μm **(A)**; 100 μm **(B)**; 50 μm **(C)**; 30 μm **(D,E)**.

**Figure 5:**
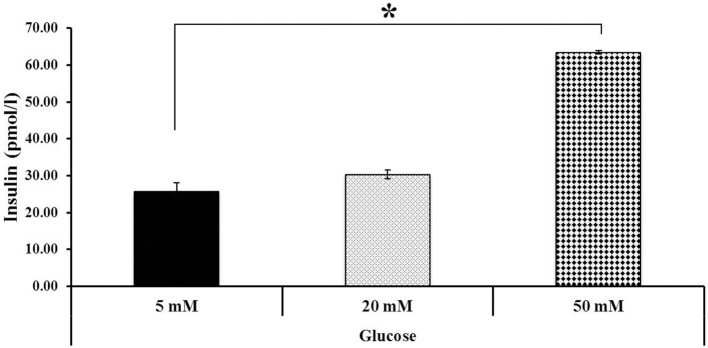
Insulin secretion at various glucose concentrations. Differentiated cells (D46) were examined for their insulin-secretion potential. The difference in insulin concentration was observed using 5, 20, and 50 mM glucose. Significant insulin concentration was observed when incubated with 50 mM glucose. The data of experiment are expressed as the mean ± SEM of three independent experiments. ^*^Values deviate significantly from corresponding 5 mM glucose (*P* ≤ 0.05).

Additionally, the differentiated hESCs (Figures 4A,B) were further tested using a dithizone (DTZ) staining. DTZ, a zinc-chelating agent, is known to selectively stain pancreatic β-cells crimson red. The cells were stained positive for DTZ (Figures 4C–E), thus confirming that our differentiation protocol could direct differentiation of hESCs into IPCs. To determine whether these cells can release insulin, we examined the insulin secretion in response to glucose by using 5, 20, and 50 mM glucose. The result revealed that the insulin levels were increased in response to higher concentration of glucose as 25.73 ± 2.41, 30.33 ± 1.14, and 63.47 ± 0.52 pmol/l to 5, 20, and 50 mM glucose, respectively. Significant insulin secretion was observed with 50 mM glucose as compared with 5 mM glucose (Figure 5). These results indicated that our hESC-DIPCs secreted insulin in response to glucose in a concentration-dependent manner.

### Insulin secretion from encapsulation of hES-DIPCs

**Figure 6:**
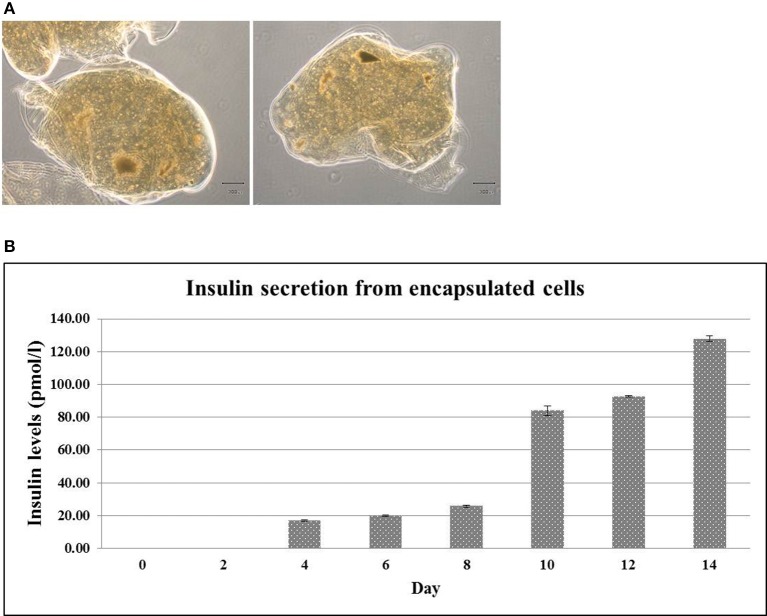
Encapsulated hES-DIPCs. Phase contrast microscopy of encapsulated hES-DIPCs **(A)**. Insulin secretion was performed by ELISA assay. Representative results of insulin secretion levels were retrieved from the encapsulated cells cultured in differentiation medium stage 3 on day 0, 2, 4, 6, 8, 10, 12, and 14 **(B)**. Scale bar = 300 μm. All values are mean ± S.E.M.

The ability of insulin releasing from encapsulated cells was further investigated. To this end, the encapsulated hES-DIPCs were formed with diameters of approximately 2,000 μm (Figure 6A). The level of insulin that presented in the culture medium was then examined by ELISA assay. It revealed that the amounts of insulin released from the encapsulated cells was increased from day 4 (16.90 ± 0.40 pmol/l) to day 14 (127.92 ± 1.58 pmol/l) under the culture condition (Figure 6B). It should be noted that the encapsulated cells possess the function of insulin secretion in a time-dependent manner.

### Fasting blood sugar (FBS) levels and body weight in diabetic mice either untreated or treated with hES-DIPCs or encapsulated hES-DIPCs injection after 70 days of treatment

**Figure 7:**
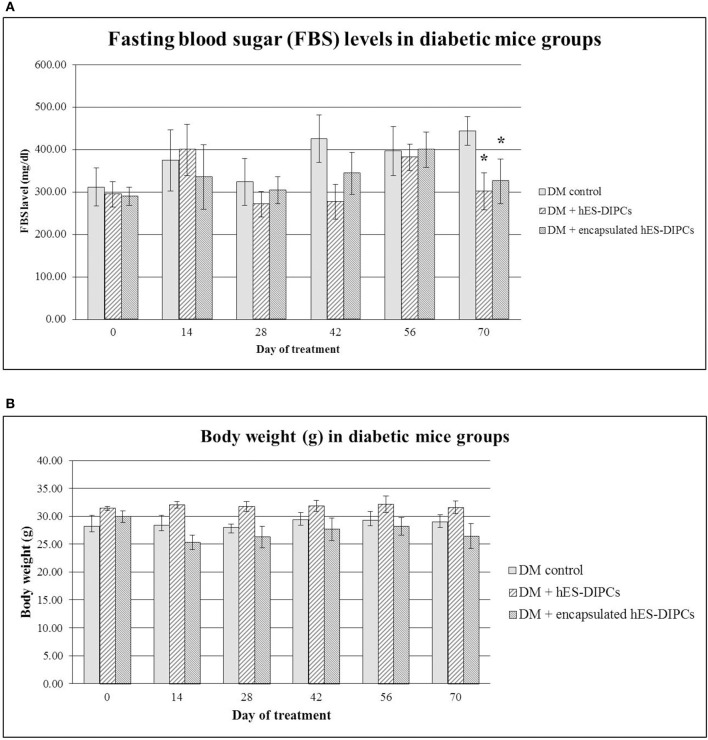
Fasting blood sugar (FBS) levels **(A)** and body weight **(B)** in diabetic mice either untreated or treated with hES-DIPCs or encapsulated hES-DIPCs. All values are mean ± S.E.M. ^*^Values deviate significantly from corresponding diabetic control mice (*P* ≤ 0.05).

FBS and body weight in diabetic mice either untreated or treated with hES-DIPCs or encapsulated hES-DIPCs injection are shown in Figures 7A,B, respectively. FBS levels at the end of experiment were significantly sustained in diabetic mice treatment with both hES-DIPCs and encapsulated cells injection groups compared to diabetic control group. In addition, there was no significant effect on body weight after treatment in three groups.

### Inflammatory evaluation (mouse IL-1β concentration) in diabetic mice either untreated or treated with hES-DIPCs or encapsulated hES-DIPCs injection after 70 days of treatment

**Figure 8:**
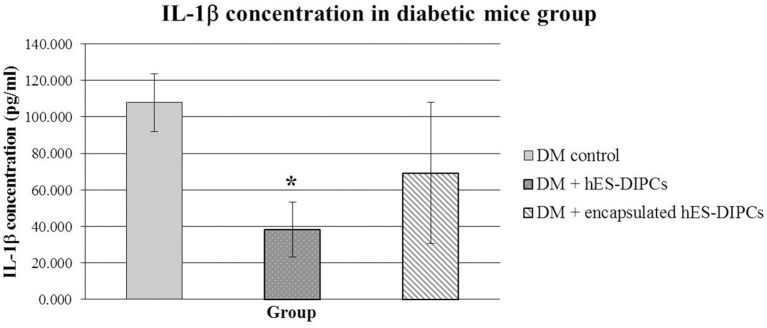
Mouse IL-1β concentration in diabetic mice either untreated or treated with hES-DIPCs or encapsulated hES-DIPCs injection. The amounts of IL-1β (mean ± SEM) are expressed as pg/ml. ^*^Values deviate significantly from corresponding diabetic control mice (*P* ≤ 0.05).

The inflammatory activity of injection of hES-DIPCs or encapsulated hES-DIPCs cells was evaluated by an ELISA assay, as shown in Figure 8. The control mice are included. In the mouse IL-1β evaluation at the end of experiment, the hES-DIPCs injection showed significant decrease compared to the diabetic control group. However, the IL-1β level was also declined in diabetic mice injected with the encapsulated cells when compared to the control group. This data suggested that both hES-DIPCs and encapsulated hES-DIPCs cells down regulated the expression of inflammatory cytokine level *in vivo*.

## Discussion

In the present protocol, a combination of various factors during differentiation was modified in order to develop more efficient condition for achieving IPCs under hypoxic environment. In this regard, the hESCs were cultured and induced toward IPCs under hypoxia (5% O_2_) throughout the works. Hypoxic culture has been shown to enhance EB formation of hESCs (Ezashi et al., 2005). Some reports have demonstrated that hypoxia exposure promoted the differentiation of chondrogenic, endothelium or neuronal from hESCs (Koay and Athanasiou, 2008; Francis and Wei, 2010; Prado-Lopez et al., 2010). Previous study has shown that the hypoxia and HGF treatment enhance the differentiation of umbilical cord blood-derived stromal cells into IPCs (Sun et al., 2015). Additionally, hypoxic conditions promoted the differentiation and depressed the self-renewal of metanephrogenic mesenchymal stem cells (Liu et al., 2017). In contrast, there is evidence that oxygen influences the different cell subsets during pancreatic development. Endocrine cells are derived from pancreatic progenitors under hypoxic condition present at early stages of development. Indeed, they progressively increased their numbers by proliferation of differentiated cells upon vascularization with a consequent increase in the availability of oxygen (Fraker et al., 2009).

To achieve hES-DIPCs induction, the growth factors were selected according to the protocols that had been reported to derive IPCs with some modifications (Wong, 2011). In this study, the IPCs were induced to differentiate from hESCs in a manner that mimics differentiation *in vivo*. The differentiation stages range from starting with definitive endoderm (DE) induction, pancreatic endoderm formation, pancreatic endocrine induction and subsequently to islet-like clusters (ILCs) maturation.

The DE is the embryonic germ layer which can give rise to the pancreas (Docherty et al., 2007). Differentiation of hESCs into IPCs was also achieved by EBs formation and the DE protocol (Wei et al., 2013). The EBs consist of 3 germ layers; ectoderm, mesoderm and endoderm layers. Therefore, we need to achieve EBs formation in order to gain the DE cells. Although, it has been demonstrated that bFGF, activin A and endothelial growth factor (EGF) treatment of hESCs could support the progression of pancreas-committed cells (Jiang et al., 2007; Xu et al., 2011; Tang et al., 2012; Wei et al., 2013; Raikwar et al., 2015). However, in this study, we aimed to use less growth factor as it is possible, therefore, the EBs formation were formed within hanging drops in the presence of activin A without both bFGF and EGF. Under this condition, high yield and healthy EBs were obtained. We observed that these EBs expressed *Nestin, Hnf3*β, Nkx6.1 transcripts. The *Nestin* is known as an important gene in pancreatic and neuronal developments. In addition, this gene expressed in both neuronal and pancreatic β-cell progenitors (Zulewski et al., 2001). The *Hnf3*β gene is a critical factor in the endodermal cell lineage development. In fact, *Hnf3*β is a transcriptional regulator of *Pdx1*, which is important in the regulation of insulin gene expression and also required for the differentiation of the mature pancreas (Soria, 2001). Our study demonstrated that defined condition and activin A were necessary to generate DE in a hypoxic atmosphere.

Next, the pancreatic endoderm stage of pancreatic lineage were generated from DE differentiation by combination of EGF, bFGF, noggin, betacellulin, and B27. It has been reported that the addition of bFGF might allow the development of pancreatic endoderm, while EGF was added to allow the growth and differentiation of pancreatic epithelial cells generated from the hESC-derived definitive endoderm (Jiang et al., 2007). In addition, the combination of bFGF and EGF has been shown to expand and promote IPCs differentiation from human bone marrow-derived stem cells (Tang et al., 2012). The selection of noggin was performed to promote differentiation of definitive endoderm to pancreatic lineage. Our findings demonstrated the presence of the islet cell precursor according to the mRNA expression of *Nestin, Ngn3, NKx6.1, Hnf36, GLUT2, and Insulin*. During early stage of IPCs differentiation from hESCs, a marker of pancreatic endocrine progenitor and neural stem cells, nestin, was observed in these cells. The expression of *nestin* in these cells has been noted to play a role in the development of pancreatic endocrine cells. The nestin-expressing cells might represent the precursors for neogenesis of pancreatic endocrine cells (Hunziker and Stein, 2000). These data also supported by the works in mESCs and hESCs which could generate the IPCs from nestin-positive cells (Lumelsky et al., 2001; Mao et al., 2009; Wei et al., 2013). The *Ngn3* transcripts were highly expressed at 21 days of the differentiation process (pancreatic endoderm induction). The expression of *Ngn3* is required for the specification of a common precursor for the four pancreatic endocrine cell types (Gradwohl et al., 2000; Collombat et al., 2006). It has been hypothesized that *Ngn3* is involved in activating the expression of *BETA2* at an early stage of islet cell differentiation through the E boxes in the *BETA2* promoter (Huang et al., 2000). This indicated that the addition of stage I differentiation cocktail allowed the growth and differentiation of the hESC-derived definitive endoderm.

Further stage of differentiation is transition of pancreatic endoderm into pancreatic endocrine stage. This step was carried out by stage II differentiation cocktail. In the absence of bFGF environment, it resulted to facilitate further stage of pancreatic development (pancreatic endocrine). At this development period, the results demonstrated the peak expression of *Pdx1, NeuroD1* transcripts compared with other stages. In addition, the expression of *Hnf36* and *Nkx6.1* transcripts was increased while Ngn3 was decreased at stage II cells compared with stage I cells.

Pdx1 is required during mid-pancreatic development for cellular differentiation. Depletion of *Pdx1* gene expression could inhibit the next development phase, the onset of acinar and islet development are blocked (Hale et al., 2005). It should be noted that endogenous *Pdx1* expression is likely to maintain the insulin-producing function (Imai et al., 2005). It has been reported that the addition of noggin was further promoted differentiation of DE to pancreatic lineage by enhancing *Pdx1* gene expression (Jiang et al., 2007).

Our results also shown that *Ngn3* is downregulated during the transition of the endocrine cell precursor into complete islets, expression of *Nkx6.1* is upregulated. It has been demonstrated that ectopic *Nkx6.1* expression is induced in *Foxa2* (*Hnf3*β) positive endodermal cells that express exogenous *Pdx1* (Pedersen et al., 2005). Moreover, recent report has shown that synergistic expression of *NeuroD1* and *Pdx1* might be crucial for maintenance of the property of IPCs derived from ESCs (Saitoh et al., 2007). This data indicates that the expression of *Nkx6.1, NeuroD1*, and *Pdx1* play a role in the formation of pancreatic β-cells.

In order to promote the mature pancreatic β-cells (stage III), the expression of a key transcription factors should be achieved *in vitro*. Additionally, the candidate factors are required to drive the maturation of progenitors. As demonstrated in our study, after pancreatic endocrine cells were exposed to the combination of nicotinamide, IGF II, betacellulin and HGF, the induction of an important transcription factors could be reached for ILCs development. There is evident showing that the addition of nicotinamide and IGF-II to induce islet-like clusters (ILCs) maturation (Jiang et al., 2007). Moreover, it was shown that the addition of betacellulin and nicotinamide sustain *Pdx1* expression and induced pancreatic β-cells differentiation under *in vitro* conditions (Cho et al., 2008). Furthermore, several studies demonstrated that HGF might be of clinical value for *in vitro* expansion of human adult beta cells (Vasavada et al., 2006; Alismail and Jin, 2014). Recently, Zhan et al. have successfully induced rat pancreatic ductal epithelial cells (PDECs) to differentiate into IPCs with HGF (Zhan et al., 2009).

In this work, the cells at the end of differentiation stage expressed the peak of the keys pancreas-related genes including *GLUT2* and *insulin*. In addition, the highest expression of *Nkx6.1* transcripts was also observed. The *Nkx6.1* is a homeobox gene presented in differentiated β-cells. Interestingly, *Nkx6.1* is also restricted to β-cells and some neurons (Docherty et al., 2007). This finding suggests the presence of a common progenitor of neurons and insulin-positive cells during *in vitro* differentiation (Soria, 2001). Moreover, the IPC derived from induced pluripotent stem cells could be enhanced by overexpression of Pdx1 and Nkx6.1 factors (Walczak et al., 2016). In the present study, the expression of *Nkx6.1* was detected starting at DE formation stage and then increased until the later stages of differentiation. Our result also revealed the expression of *Pdx1* gene expression at ILCs maturation stage. As described by Cho et al., since the expression of *Pdx1* is crucial for *insulin* gene expression, therefore, the addition of betacellulin was selected to sustain *Pdx1* gene expression through the differentiation of IPCs (Cho et al., 2008).

Moreover, the *Ngn3* transcripts was also obtained at the stage III cells. It has been demonstrated that Ngn3 deficiency results in reduced branching and enlarged pancreatic duct-like structures (Magenheim et al., 2011). Similarly, it has been demonstrated that *Ngn3* and *NeuroD1/BETA2* can drive the early differentiation of islet cells (Schwitzgebel et al., 2000). *Ngn3* has been shown to induce the expression of *BETA2* (*NeuroD*), a transcription factor implicated in the insulin gene expression and in islet cells differentiation (Soria, 2001). However, the *Nestin* and *Hnf3*β expressions were also observed in stage III cells representing the presence of the immature stage of pancreatic development.

To confirm the accomplishment of hES-DIPCs induction, the key islet-specific hormone insulin was determined by immunofluorescence staining. The insulin protein expression is represented by the presence of C-peptide; a genuine marker of *de novo* insulin production. The results demonstrated the high expression of C-peptide protein in these cells. Therefore, this work achieves to generate hES-DIPCs *in vitro*. However, the islet-specific hormones somatostatin and glucagon were also detected a long with some nestin positive cells. These indicated that there are other islet cell types rather than mature β-cells presented in this culture. In this regard, the further improvement of protocol is needed to yield pure β-cell population.

More evidence to support the hES-DIPCs achievement are DTZ-staining of the cells. Therefore, the clusters at days 46 of differentiation were subjected to the DTZ-stain examination. It has been demonstrated that DTZ stain could be applied for the detection of ES-derived IPCs. The zinc-chelating agent DTZ is known to selectively stain pancreatic β-cells, which contain relatively high levels of zinc (Shiori et al., 2005; Baharvand et al., 2006; Marappagounder et al., 2013). Our data showed the presence of DTZ-positive cellular clusters in ILCs population indicating the successful hES-DIPCs differentiation.

Further *in vitro* functional characterization of hES-DIPCs is also required to verify the achievement of hES-DIPCs creation. To end this, the insulin secretion from stage III cells was assessed by glucose stimulation. This experiment revealed that the hES-DIPCs could secrete insulin in a glucose-dependent manner. However, the level of insulin secretion by our hES-DIPCs was considerably lower than the natural human pancreatic islets. As reviewed by Naujok and colleagues, the lower insulin secretory capacity of these cells may be due to the present differentiation protocol does not provide a sufficient number of IPCs meeting the functional criteria of genuine β-cells. It became apparent that the cells obtained were heterogeneous population of endocrine cells. However, these problems has been overcome through an *in vivo* environment facilitated the extensive maturation into islet cells with a robust insulin and C-peptide release (Naujok et al., 2011).

The clinical application capacity of hES-DIPCs for diabetes treatment is another crucial issue that need to be explored. Most reported strategies have focused on permitting the reversal of hyperglycemia in diabetic models by replacing β-cells via cells transplantation. For example, Shim and co-workers have reported that transplantation of hESC-derived Pdx1-positive cells was further produced the cells that express insulin, proinsulin, C-peptide and glucagon. These cells were then resulted in amelioration of hyperglycaemia and weight loss in streptozotocin-treated diabetic mice (Shim et al., 2007). Interestingly, Jiang and colleagues have used a modified approach in serum-free culture medium to induce hESCs to differentiate into functional IPCs. The IPCs were then transplanted under the renal capsules of diabetic nude mice. The results showed that these cells could rescue the hyperglycemia phenotype in thirty percent of the transplanted nude mice and maintained it for more than 6 weeks (Jiang et al., 2007). However, it was shown that the expression of human leukocyte antigens (HLA) was considerably inducible to a higher level after differentiation (Draper et al., 2002; Drukker et al., 2002; van der Torren et al., 2017). Thus, it is possible that the recipient immune system rejected the transplanted hESC-derived cells. As mentioned, the immune isolation by encapsulation can be applied to protect implanted-cells against antibodies and cytotoxic cells of the host immune system (Paredes Juárez et al., 2014). Also it has been shown that alginate encapsulant allows for transplantation of cells in the absence of immunosuppression (de Vos et al., 2006; O'Sullivan et al., 2011).

Therefore, in this work, transplantations of hES-DIPCs and encapsulated hES-DIPCs in diabetic animal models were investigated in order to examine the *in vivo* function of the cells. To complete this, first, encapsulated hES-DIPCs were tested the ability of insulin secretion out of the encapsulated particle. The results noted that the encapsulated cells possess the function of insulin secretion in a time-dependent manner. Next, the hES-DIPCs and encapsulated hES-DIPCs were used to transplanted under subcutaneous of STZ-induced diabetic mice. Applying the encapsulated cells for injection, these encapsulated cells were firstly evaluated the insulin release from alginate encapsulation. The data thus indicated that the encapsulated cells release insulin into the medium at time point following incubation. After the cells injection process, FBS levels of all the treated mice showed a significant maintain after 70 days of treatment compared to the control, suggesting that the injected-cells was responsible for the restoration of glucose levels. In addition, the excellent health and pelage status were observed in both hES-DIPCs and encapsulated hES-DIPCs injection mice. Interestingly, the hES-DIPCs injection also showed significantly decreased in mouse IL-1β concentration when compared to the control. Although the concentration of mouse IL-1β in diabetic mice injected with encapsulated cells was higher than the hES-DIPCs injection, there was no statistically significant different. It has been established that IL-1β was produced by activated macrophages and monocytes, which correlated with the inflammatory process (Afonina et al., 2015). These data also proved that the both hES-DIPCs and encapsulated hES-DIPCs cells could sustain the levels of blood sugar as well as decrease inflammatory cytokine level. These findings also suggested that the encapsulated hES-DIPCs may involve the declination of immune rejection possibility at post-transplantation.

It has also been suggested that the development and progression of diabetic complications are associated with inflammation, which produces the inflammatory mediators, such as pro-inflammatory cytokines (Navarro, 2005; Navale and Paranjape, 2013). In the impaired glucose tolerance (IGT) subjects, hyperglycemia was shown to increase circulating cytokine concentrations by an oxidative mechanism (Esposito et al., 2002). According to Garcia et al., inflammation markers are increased in diabetic patients and also can predict cardiovascular risk as they are associated with endothelial dysfunction (Garcia et al., 2010). Nevertheless, insulin has been considered a regulator of inflammatory and immune responses in which it can attenuate inflammation and regulate immune reactions. Intensive insulin therapy is also maintained the inflammatory reaction balance, which significantly decrease pro-inflammatory cytokine levels and increase anti-inflammatory cytokine levels (Deng and Chai, 2009).

Due to its limit the therapeutic approach in diabetic models, the alginate encapsulation is partly responsible for the host immune reaction against encapsulated cells. Moreover, the impaired cell survival is caused by the high immunogenicity of unpurified alginates (Langlois et al., 2009). Furthermore, Arnush et al. have shown that the expression of IL-1β results in the inhibition of β-cells function (Arnush et al., 1998). Thus, the absence of encapsulated cells injection treatment to prevent diabetes complications is also influenced by the depletion of insulin secretion. These results suggest that the purity of the alginate type and the encapsulation process should be warranted to evaluate such potentially available therapeutic approach.

## Conclusions

**Figure 9:**
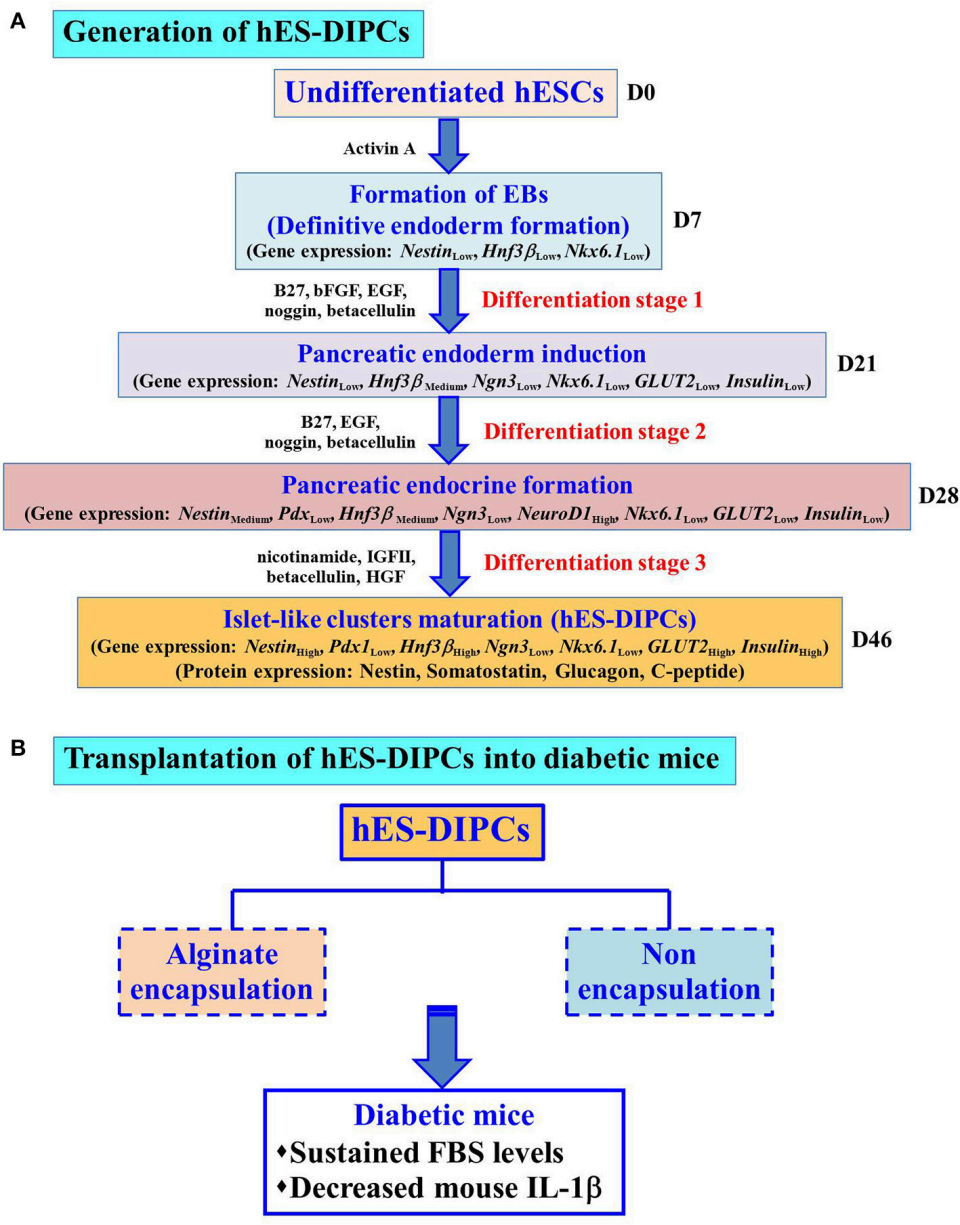
Hallmark of the procedures and findings of this study. Schematic representation of the differentiation protocol to generate hES-DIPCs with hypoxic treatment. These cells were indicated possible differentiation range from definitive endoderm induction, pancreatic endoderm formation, pancreatic endocrine induction and subsequently to islet-like clusters maturation. Gene expression levels (*Nestin, Pdx1, Hnf3*β, *Ngn3, NeuroD1, Nkx6.1, GLUT2*, and *Insulin*) among the cells at different stages (D7, D21, D28, and D46) were analyzed. The results demonstrate the expression of *GLUT2* and *Insulin* was highly induced at D46 (Islet-like clusters maturation stage). Also, the cells at D46 exhibit the protein expression of nestin, somatostatin, glucagon and C-peptide **(A)**. These differentiated cells, namely hES-DIPCs were then determined their functions *in vivo* The hES-DIPCs and encapsulated hES-DIPCs were subjected to transplanted into STZ-induced diabetic mice. These cells possessed the ability to sustain fasting blood sugar (FBS) levels as well as decrease inflammatory cytokine (mouse IL-1β) levels **(B)**.

We have propose a new differentiation protocol to generate the IPCs from hESCs under hypoxic condition. The hES-DIPCs showed characteristics of IPCs *in vitro* in both mRNA and protein levels. In addition, IPCs exhibited DTZ-positive cellular clusters and insulin secretion in a glucose-dependent manner. The application of these findings is generation of a large number of hES-DIPCs *in vitro*. Moreover, both hES-DIPCs and encapsulated hES-DIPCs possessed the ability to prevent hyperglycemia condition as well as decrease inflammatory cytokine level in STZ-induced diabetic mice. In addition, the implication of this work is therapeutic value in type I diabetes treatment in the future. The application for type II diabetes treatment remain to be investigated. Further additional factors and mechanisms are also needed to enrich the IPCs maturation and yield pure β-cells. The hallmark of this work is depicted in Figure 9.

## Author contributions

PR: performed the experiments and wrote part of the manuscript; CD: helped in review and preparation of the manuscript; WL: wrote the grant, instructed and solved the problems of the experiments, wrote parts of the manuscript and completed the preparation of the manuscript.

### Conflict of interest statement

The authors declare that the research was conducted in the absence of any commercial or financial relationships that could be construed as a potential conflict of interest.

## Abbreviations

hESCs: human embryonic stem cells
hES-DIPCs: hESC-derived insulin-producing cells
EBs: embryoid bodies
ILCs: islet-like clusters
DM: Diabetes mellitus
IPCs: insulin-producing cells
encapsulated hES-DIPCs: encapsulated human embryonic derived insulin producing cells
hFF: human forskin fibroblast
KO-DMEM: knockout Dulbecco's modified Eagle's medium
KO-SR: knockout serum replacement
bFGF: basic fibroblast growth factor
PCR: polymerase chain reaction
RT: reverse transcription
PBS: phosphate buffer saline
DAPI: diamidino-2-phenylindole
DTZ: Dithizone
DMSO: dimethylsulfoxide
HBSS: Hank's balanced salt solution; Krebs-Ringer bicarbonate HEPES (KRBH) buffer
ELISA: enzyme-linked immunosorbent assay
SUT: Suranaree University of Technology
STZ: Streptozotocin
SC: subcutaneously
FBS: fasting blood sugar
IL-1β: Interleukin 1 beta
MDD: mean minimum detectable dose
EGF: endothelial growth factor
HLA: human leukocyte antigens
IGT: impaired glucose tolerance.
